# Failure to upregulate cell surface PD-1 is associated with dysregulated stimulation of T cells by TGN1412-like CD28 superagonist

**DOI:** 10.4161/mabs.29758

**Published:** 2014-10-30

**Authors:** Thilipan Thaventhiran, Naif Alhumeed, Han XA Yeang, Swaminathan Sethu, Jocelyn S Downey, Ahmad F Alghanem, Adedamola Olayanju, Emma L Smith, Michael J Cross, Steven D Webb, Dominic P Williams, Adrian Bristow, Christina Ball, Richard Stebbings, Jean G Sathish

**Affiliations:** 1 Medical Research Council Centre for Drug Safety Science and Department of Molecular and Clinical Pharmacology; University of Liverpool; Liverpool, UK; 2 Rheumatology Division; Washington University School of Medicine; Saint Louis, MO USA; 3 School of Bio Sciences & Technology and Centre for Biomaterials Science & Technology; VIT University; Vellore, India; 4 St. Edmund's College; University of Cambridge; Cambridge, UK; 5 National Institute for Biological Standards and Control; Potters Bar; Hertfordshire, UK

**Keywords:** TGN1412, T cells, CD28 superagonist, immunostimulatory biologics, PD-1

## Abstract

The CD28 superagonist (CD28SA) TGN1412 was administered to humans as an agent that can selectively activate and expand regulatory T cells but resulted in uncontrolled T cell activation accompanied by cytokine storm. The molecular mechanisms that underlie this uncontrolled T cell activation are unclear. Physiological activation of T cells leads to upregulation of not only activation molecules but also inhibitory receptors such as PD-1. We hypothesized that the uncontrolled activation of CD28SA-stimulated T cells is due to both the enhanced expression of activation molecules and the lack of or reduced inhibitory signals. In this study, we show that anti-CD3 antibody-stimulated human T cells undergo time-limited controlled DNA synthesis, proliferation and interleukin-2 secretion, accompanied by PD-1 expression. In contrast, CD28SA-activated T cells demonstrate uncontrolled activation parameters including enhanced expression of LFA-1 and CCR5 but fail to express PD-1 on the cell surface. We demonstrate the functional relevance of the lack of PD-1 mediated regulatory mechanism in CD28SA-stimulated T cells. Our findings provide a molecular explanation for the dysregulated activation of CD28SA-stimulated T cells and also highlight the potential for the use of differential expression of PD-1 as a biomarker of safety for T cell immunostimulatory biologics.

## Abbreviations

APC: antigen presenting cell
CCR5: C-C chemokine receptor type 5
CD28SA: CD28 superagonist
CK2: casein kinase 2
CTLA-4: cytotoxic T-Lymphocyte Antigen 4
IFNγ: interferon gamma
IL-2: interleukin 2
LAG-3: Lymphocyte-activation gene 3
LFA-1: lymphocyte function-associated antigen 1
MFI: mean fluorescence intensity
PBMC: peripheral blood mononuclear cells
PD-1: programmed cell death protein 1
PD-L1: programmed cell death-ligand 1
PTEN: phosphatase and tensin homolog
S-phase: synthesis phase
TCR: T cell receptor
T_EMs_: effector memory T cells
TIM-3: T cell immunoglobulin mucin 3

## Introduction

Immunostimulatory antibodies are in clinical trials for a variety of indications,^1^ particularly in eliciting anti-tumor responses but they come with a risk of serious adverse effects such as systemic induction of pro-inflammatory cytokines (cytokine storm) and organ-specific autoimmunity.^2^ In 2006, a phase I first-in-man dose-escalation trial of TGN1412, a humanized CD28-specific superagonistic mAb originally intended for the treatment of B cell chronic lymphocytic leukemia and rheumatoid arthritis, caused severe immune-mediated toxicity in a cohort of healthy volunteers.^3^ The unforeseen biological action in humans included substantial proliferation and extravasation of T cells, and a life-threatening cytokine storm with highly elevated levels of various pro-inflammatory cytokines.^4^

Physiological T cell activation occurs when the T cell receptor (TCR) is engaged by an antigen-bearing MHC molecule on the surface of antigen presenting cells (APCs) and this represents the first signal for T cell activation. Co-stimulatory receptors such as CD28 can act as secondary signals to amplify this first signal. In general, CD28 ligation in the absence of TCR engagement has no functional effect on T cells; however, CD28SA antibodies can activate T cells without concomitant TCR engagement.^5^

Activation of T cells through TCR triggering and CD28 engagement leads to downregulation of cell surface TCR, expression of molecules such as lymphocyte function-associated antigen-1 (LFA-1) and C-C chemokine receptor type 5 (CCR5), synthesis of interleukin (IL)-2 and T cell proliferation. Importantly, the activation of T cells is regulated by the expression and function of co-inhibitory receptors such as PD-1, T cell immunoglobulin mucin 3 (TIM-3), Lymphocyte-activation gene 3 (LAG-3), and Cytotoxic T-lymphocyte-associated protein 4 (CTLA-4).^6^ These co-inhibitory receptors prevent excessive T cell activation by attenuating the activation signals initiated by T cell stimulatory receptors.^7^ The PD-1 co-inhibitory receptor has been shown to be a powerful negative regulator of activated T cells.^8^ In the absence of PD-1-mediated signals, there is an increased propensity for T cells to expand with stronger accompanying inflammatory cascades.^9^ The excessive T cell activation observed with CD28SA-stimulated T cells suggests a dysregulation of the inhibitory inputs to these T cells. We hypothesized that a lack of inhibitory inputs from the PD-1 pathway could contribute to the uncontrolled proliferation and cytokine release of CD28SA-activated T cells. In this study, we characterized the dysregulated T cell phenotype and function of CD28SA activated T cells and show that these T cells lack regulation mediated by the PD-1 pathway.

## Results

### Dysregulated T cell function induced by CD28SA stimulation

Ligation of the TCR/CD3 complex activates T cells and co-engagement of CD28 with TCR/CD3 enhances this response, while in general, ligation of CD28 in isolation with conventional monoclonal antibodies does not stimulate T cells. To assess the effects of CD28SA-mediated T cell stimulation, we activated human T cells with either NIB1412 (CD28SA), or anti-CD3. Previous studies have identified CD4^+^ effector memory T cells (T_EMs_) as the primary responders to CD28SA stimulation,^10,11^ and therefore we mainly focused on T_EMs_ for phenotypic and functional assessments.

**Figure 1.**
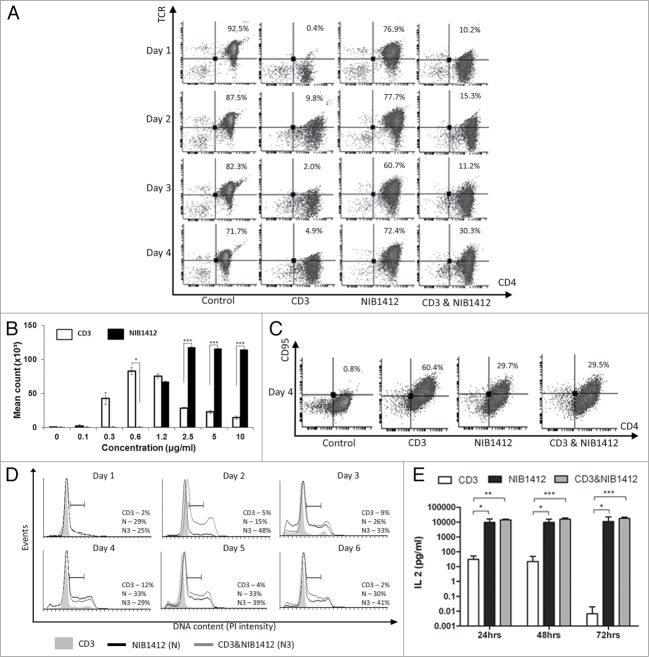
Dysregulated T cell function induced by CD28SA stimulation. (**A**) Human CD4^+^ T_EMs_ were stimulated for 1 to 4 d with plate-bound anti-CD3 mAb (CD3, 5 μg/ml); NIB1412 (NIB1412, 10 μg/ml); anti-CD3 mAb and NIB1412 (CD3 and NIB1412); control category included cells without any treatment (Control). Cells were harvested at indicated time points and stained with fluorochrome-conjugated anti-CD4 and anti-TCR antibodies followed by flow cytometric analysis. Population of CD4^+^TCR^+^ cells are shown in the upper right quadrant as percentages of total T cells. Results are representative of four independent experiments. (**B**) Human CD4^+^ T_EMs_ cells were stimulated with the indicated concentrations of plate-bound antibodies and proliferation was measured three days post-activation by ^3^H-labeled thymidine incorporation. The vertical axis represents mean cpm ± SD from triplicate wells. The data are representative of four independent experiments, (*P < 0.05, ***P < 0.001; unpaired *t* test). (**C**) Cell surface staining of human CD4^+^ T_EMs_ stimulated for 4 d with 5 μg/ml of plate-bound anti-CD3 mAb and/or 10 μg/ml of NIB1412. The percentages of the CD4^+^ CD95^+^ cells are shown in the upper right quadrant. Results are representative of three independent experiments (**D**) Human CD4^+^ T_EMs_ were stimulated for 1 to 6 d, fixed with ice cold 70% ethanol, stained with propidium iodide and cells in S-phase were quantified by flow cytometry. Results are representative of at least four independent experiments. (**E**) IL-2 concentrations in the supernatants from human CD4^+^ T_EMs_ stimulated for 24, 48 and 72 h were determined by enzyme-linked immunosorbent assay (ELISA). The IL-2 titers from four independent experiments (mean ± SD of replicate samples) are expressed as picograms per mL on a log_10_ scale. (*p < 0.05, **p < 0.01, ***p < 0.001; two-way ANOVA).

Phosphorylation of the TCR/CD3 complex upon T cell activation results in a rapid downregulation of the complex,^12^ to allow for the desensitization of the stimulated cell. We have shown that NIB1412-activated T_EMs_ maintain elevated TCR expression levels of up to 80%, similar to that of unstimulated cells, while anti-CD3 stimulated T_EMs_ rapidly reduce TCR expression upon activation and maintain negligible surface expression throughout day 1 to 4 (**Fig. 1****A**). Our results also show that NIB1412-stimulated T_EM_ cells showed greater proliferation at antibody concentrations of 2.5, 5 and 10 μg/ml, displayed a higher maximum proliferative capacity compared with anti-CD3-stimulated T cells (**Fig. 1****B**), and also express lower levels of the prototypical death receptor Fas (CD95) (**Fig. 1****C**).

**Figure 2.**
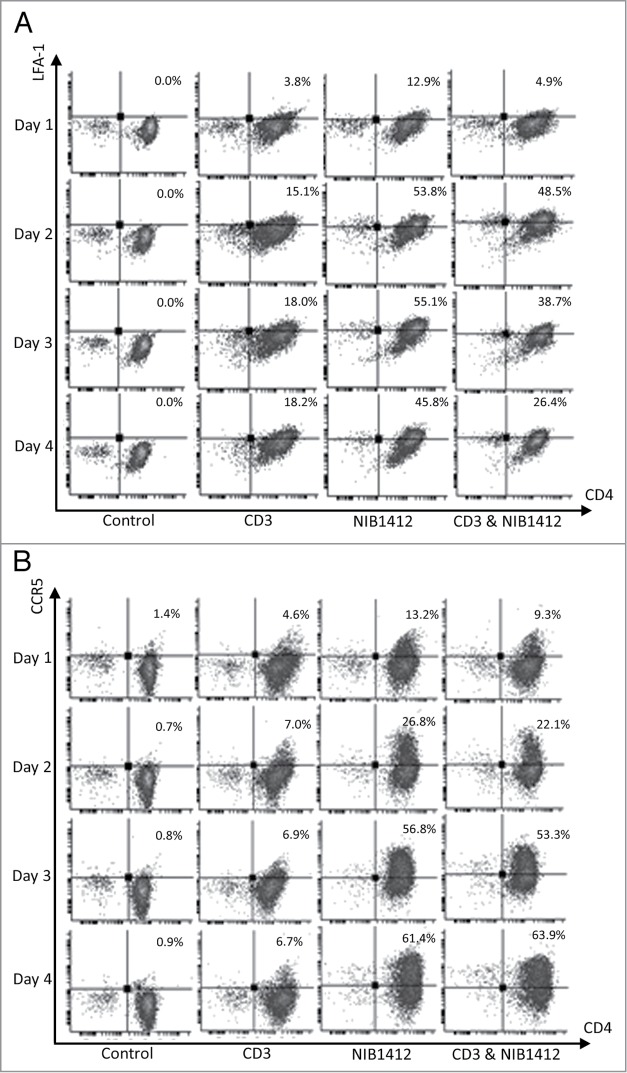
Enhanced cell surface expression of LFA-1 and CCR5 on CD28SA-activated CD4^+^ effector memory T cells. Human CD4^+^ T_EMs_ were stimulated for 1 to 4 d with plate-bound anti-CD3 mAb (CD3, 5 μg/ml); NIB1412 (NIB1412, 10 μg/ml); anti-CD3 mAb and NIB1412 (CD3&NIB1412); control category included cells without any treatment (Control). Cells were harvested at indicated time points and stained with fluorochrome-conjugated anti-CD4 and anti-LFA (**A**) or anti-CD4 and anti-CCR5 (**B**) antibodies followed by flow cytometric analysis. (**A**) Population of CD4^+^LFA^+^ cells are shown in the upper right quadrant as percentages of total T cells. The cells are shown as percentages of total T cells in the upper right quadrant. (**B**) Population of CD4^+^CCR5^+^ cells are shown in the upper right quadrant as percentages of total T cells. Results are representative of four independent experiments.

**Figure 3.**
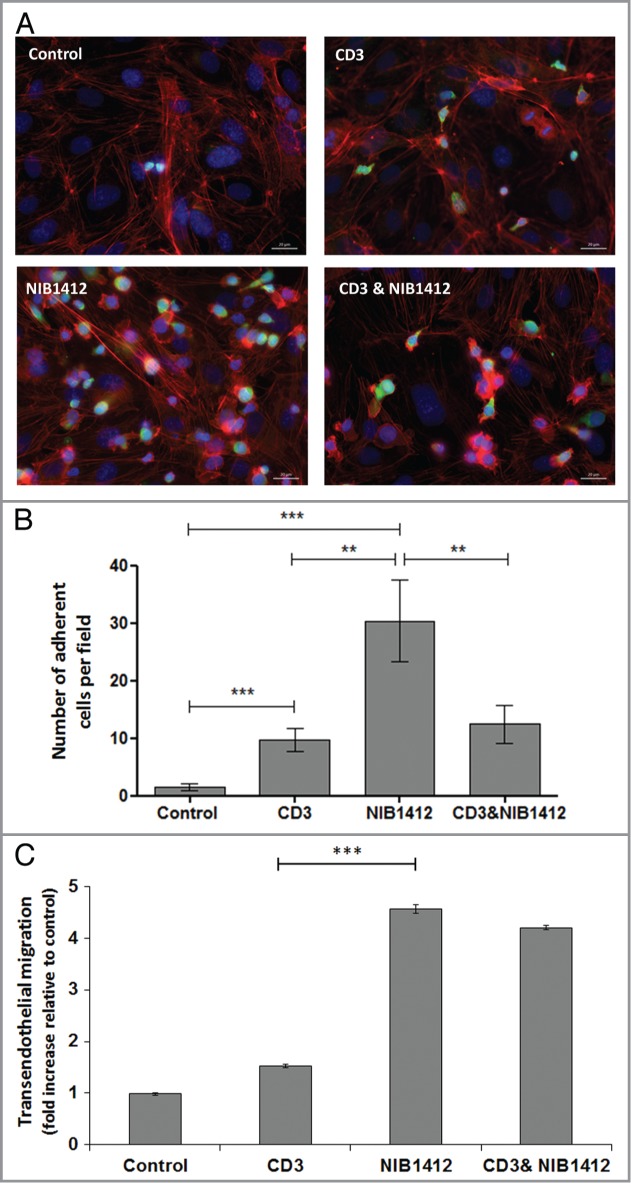
Adhesion and transmigration of CD28SA-activated CD4^+^ effector memory T cells. Human CD4^+^ T_EMs_ were stimulated with plate-bound anti-CD3 mAb (CD3, 5 μg/ml); NIB1412 (NIB1412, 10 μg/ml); anti-CD3 mAb and NIB1412 (CD3 and NIB1412); control category included cells without any treatment (Control). Cells were harvested at 48 h and labeled with CellTracker™ Green CMFDA. (**A**) Immunofluorescent microscopy of HDMEC monolayer with adherent CMFDA-labeled T_EMs_ initially stimulated with the indicated antibodies and stained for F-actin and DNA using fluorescently labeled phalloidin (red) and DAPI (blue), respectively. (Scale bar = 20 microns). Phase optics was adjusted to emphasize adherent cells. (**B**) CMFDA-labeled T_EMs_ were added to confluent HDMECs and incubated for 30 min. Cells were washed and the remaining adherent cells visualized under fluorescence microscopy. Vertical axis represents the number of adherent T_EMs_ per field (**p < 0.01; ***p < 0.001, unpaired *t* test). Results from four independent donors are represented as means ± SD (**C**) Transmigration of stimulated CD4^+^ T_EMs_ across HDMEC layer on transwell inserts. Transmigrated cells were quantified by fluorescence and the results are presented as the mean (± SD) number of transmigrated cells from 3 replicate wells per condition. Results are representative of three independent experiments (***p < 0.001; unpaired *t* test).

**Figure 4.**
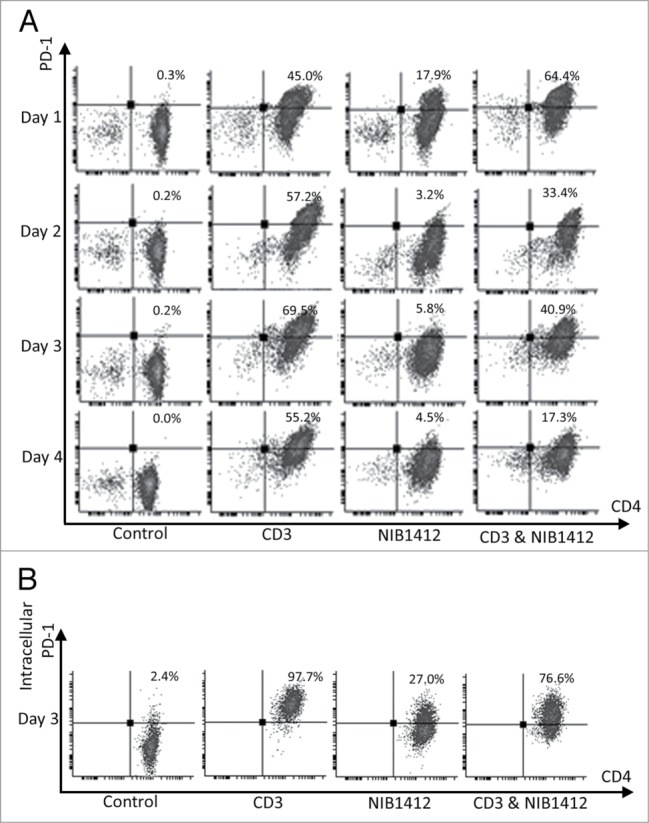
Failure of CD4^+^ effector memory T cells to upregulate cell surface PD-1 after CD28SA-activation. Human CD4^+^ T_EMs_ were stimulated for 1 to 4 d with plate-bound anti-CD3 mAb (CD3, 5 μg/ml); NIB1412 (NIB1412, 10 μg/ml); anti-CD3 mAb and NIB1412 (CD3 and NIB1412); control category included cells without any treatment (Control). **(A)** Cells were harvested at indicated time points and stained with fluorochrome-conjugated anti-CD4 and anti-PD-1 antibodies followed by flow cytometric analysis. **(B)** Cells harvested at day 3 were stained for intracellular PD-1 following fixation/permeabilization treatment. Population of CD4^+^ PD-1^+^ cells are shown in the upper right quadrant as percentages of total T cells. Results are representative of four independent experiments.

**Figure 5.**
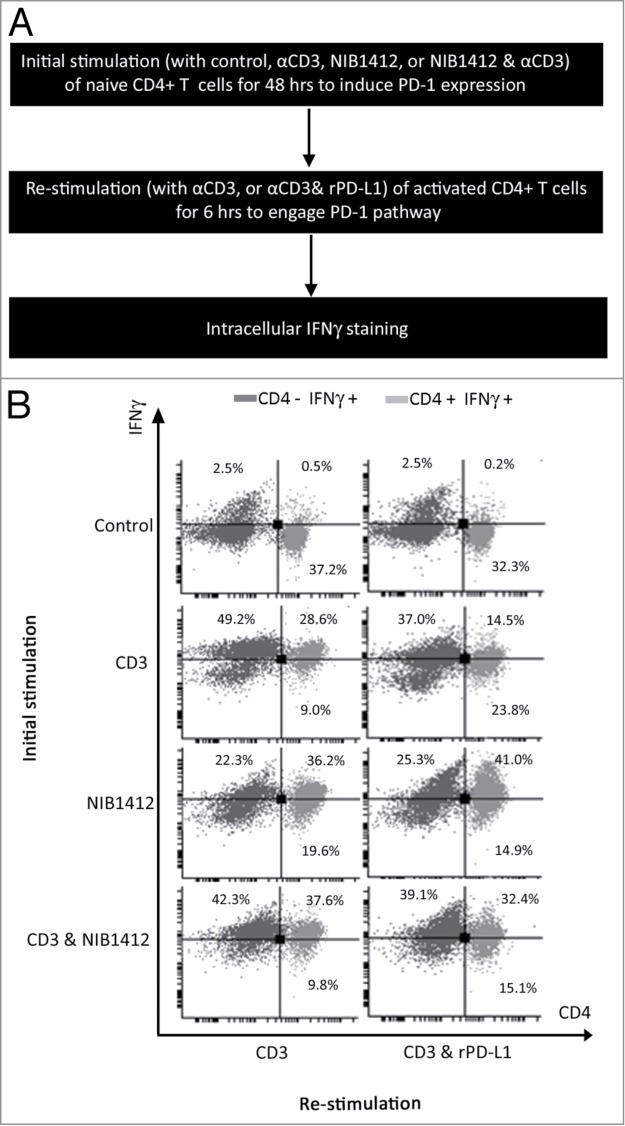
Absence of PD-1 mediated regulation of T cell function in CD28SA- stimulated T cells. (**A**) Schematic of the protocol used to investigate the functional significance of PD-1 pathway on NIB1412-activated CD4^+^ T cells. (**B**) Human PBMCs were stimulated for 48 h with plate-bound anti-CD3 mAb (CD3, 5 μg/ml); NIB1412 (NIB1412, 10 μg/ml); anti-CD3 mAb and NIB1412 (CD3 and NIB1412); control category included cells without any treatment (Control). Cells were then re-stimulated with anti-CD3 only (CD3,1 μg/ml) or with anti-CD3 and 10 μg/ml of rPD-L1 (CD3 and rPD-L1). Cells were harvested and stained with fluorochrome-conjugated anti-CD4 antibody, fixed, permeabilized, stained with fluorochrome-conjugated anti-IFNγ antibody and analyzed by flow cytometry. The CD4^+^ population is shown in light gray and the CD4^−^ population in dark gray. The percentages of the CD4^+^ IFNγ^+^ cells are shown in the upper right quadrant. Results are representative of four independent experiments.

**Figure 6.**
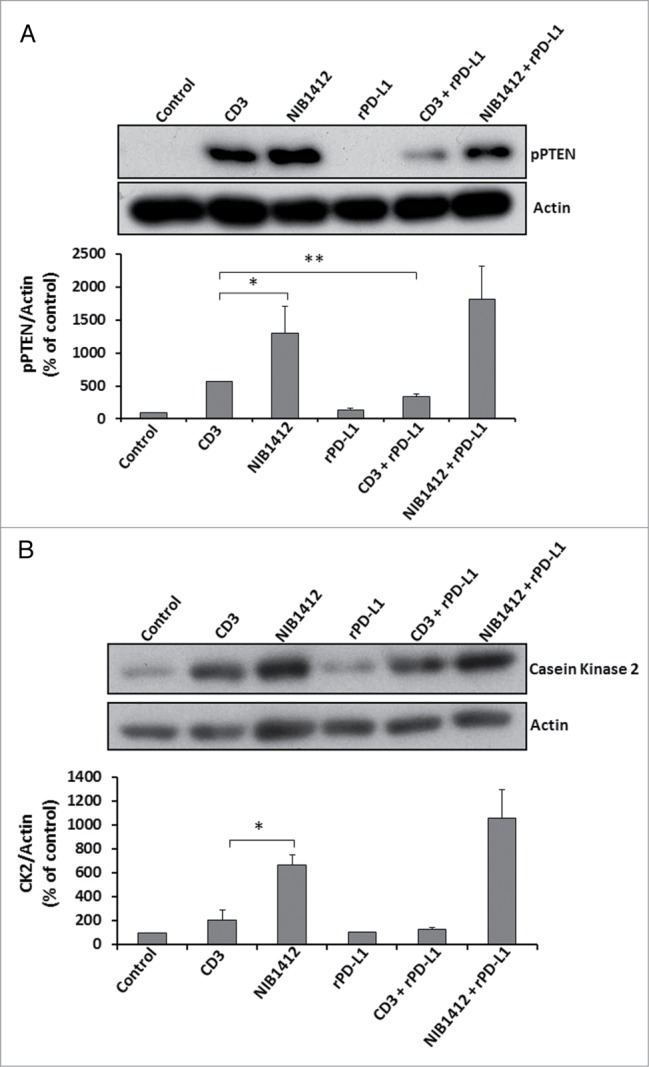
Effects of PD-1 engagement on phospho PTEN and casein kinase 2 levels in CD4^+^ effector memory T cells stimulated by anti-CD3 and CD28SA. Human CD4^+^ T_EMs_ were stimulated with anti-CD3 mAb (CD3, 5 μg/ml); NIB1412 (NIB1412, 10 μg/ml); rPD-L1 (rPD-L1, 10μg/ml); with anti-CD3 mAb and rPD-L1 (CD3 + rPD-L1) or with NIB1412 and rPD-L1 (NIB1412 + rPD-L1). At the end of 72 h, the cells were lysed and extracted protein separated by SDS-PAGE. pPTEN – S380/T382/383 (**A**) and CK2 (**B**) protein levels were assessed by immunoblotting and β-actin was used as loading control. All blots are representative of four independent experiments. pPTEN and CK2 levels were quantified by densitometry, normalized to β-actin and expressed as a percentage of pPTEN or CK2 levels in untreated cells. Data are represented as mean ± SEM of four independent experiments. Statistical analysis was performed using unpaired t test (*p < 0.05, **p < 0.01). Phospho PTEN – pPTEN; Casein Kinase 2 – CK2

To better understand the proliferative activity of stimulated T_EMs_, we determined the percentage of cells in S-phase of the cell cycle. A higher percentage of NIB1412-stimulated T_EMs_ were in S-phase throughout the measured six days. The proportion of anti-CD3-stimulated T_EMs_ in S-phase was highest on day 4 with percentages of up to 12%, which then reduced in numbers while the proportion of NIB1412-stimulated T_EMs_ in S-phase remained high at around 30%. Following day 4, the percentage of cells in S-phase remained stable in NIB1412-stimulated T_EMs_ (**Fig. 1****D**). When NIB1412 was combined with anti-CD3, the percentage of cells in S-phase peaked earlier (at day 2) and was significantly higher than the cells stimulated with either of the antibodies on their own. This result indicates that NIB1412-stimulated T_EMs_ remain in S-phase for a much longer period than anti-CD3-stimulated T cells.

IL-2 is a prerequisite growth factor for the long-term proliferation and survival of activated T cells.^13^ *In vitro* studies of TGN1412 have shown high levels of IL-2 cytokine release.^14^ In the current study, NIB1412-stimulated T_EMs_ displayed prolonged and higher IL-2 secretion of up to 25 000pg/ml, compared with ∼5000 pg/ml of IL-2 secretion by anti-CD3-stimuated T_EMs_. The anti-CD3 and NIB1412 combination-stimulated T_EM_ population also displayed high IL-2 secretion (**Fig. 1****E**). Our results show elevated IL-2 release by T_EMs_ when stimulated with NIB1412, which may contribute to the prolonged S-phase observed in the NIB1412-stimulated conditions.

### Enhanced expression of LFA-1 and CCR5 on CD28SA-activated CD4^+^ effector memory T cells

Following the TGN1412 clinical study, it was found that the lymphocytes migrated from the blood stream into organs causing significant tissue damage.^3,15^ The capability of superagonists to upregulate chemokine and adhesion receptors has not been investigated. In this study, flow cytometric analysis of the cell surface expression of LFA-1 and CCR5 revealed that a much higher percentage of NIB1412-activated T_EMs_ express LFA-1 (up to 3-fold higher) and CCR5 (up to 8-fold higher) compared to T_EMs_ that were activated with anti-CD3 mAb (**Fig. 2****A and B**). Combined anti-CD3 and NIB1412-stimulated T_EMs_ displayed an LFA-1 expression level intermediate to that of either agonist alone, while CCR5 expression was similar to that of NIB1412-stimulated T_EMs_.

### Enhanced adhesion and migration of CD28SA-activated CD4^+^ effector memory T cells

The ability of T cells to adhere and migrate along endothelial surfaces is dependent on the binding of LFA-1 on T cells to the intercellular adhesion molecule-1 (ICAM-1) expressed on endothelial cells.^16^ Since our data showed elevated LFA-1 expression on NIB1412-stimulated T_EMs_, we investigated their attachment and migratory capabilities. We show that NIB1412-stimulated T_EMs_ adhere to (**Fig. 3****A and B**) and migrate (**Fig. 3****C**) across endothelial cell layers in significantly greater numbers than anti-CD3-stimulated T_EMs_.

### Failure to upregulate cell surface PD-1 by CD28SA-activated CD4^+^ effector memory T cells

Activation of T cells via the TCR/CD3 complex leads to upregulation of cell surface PD-1. Up to 18% of NIB1412-stimulated T_EMs_ expressed PD-1, which declined to ∼3 to 6% from 48 h onwards (**Fig. 4****A**). Anti-CD3 stimulated T_EMs_ expressed high PD-1, which peaked around day 3 to about 60% and remained relatively constant up to 4 d post-activation. The proportion of PD-1^+^ T_EMs_ on day 2 post-activation was up to 17-fold higher in the anti-CD3 mAb condition compared with the NIB1412 condition (**Fig. 4****A**). From day 2 onwards, the proportion of anti-CD3-activated CD4^+^ T_EMs_ expressing PD-1 was 11- to 12-fold higher compared to those that were stimulated with NIB1412. The combination of NIB1412 and anti-CD3 generally resulted in PD-1 expression intermediate to that of stimulation with either anti-CD3 or NIB1412 (**Fig. 4****A**). Although surface staining of NIB1412-activated CD4^+^ T cells showed a negligible surface PD-1 expression, intracellular staining of these cells revealed the presence of significant levels of intracellular PD-1 (**Fig. 4****B**). Our results show that NIB1412 stimulation of T_EMs_ results in a dysregulated phenotype in terms of minimal PD-1 surface expression, which prevents the input of normal negative regulatory signals to control T cell activation.

### Absence of PD-1 mediated regulation of T cell function in CD28SA- stimulated T cells

To determine whether the absence of PD-1 expression has a functional consequence on NIB1412-activated CD4^+^ T cells, recombinant PD-L1 (rPD-L1) was used to engage the PD-1 expressed on activated T cells (shown schematically in **Figure 5****A**). The proportion of cells expressing IFNγ was decreased to half in CD4^+^ T cells that were initially stimulated with anti-CD3 and then re-stimulated with anti-CD3+rPD-L1 (**Fig. 5****B**). CD4^+^ T cells that were initially stimulated with the CD3 and NIB1412 combination were unaffected in their IFNγ secretion upon re-stimulation with anti-CD3 and rPD-L1 (**Fig. 5****B**). These results suggest that the presence of surface PD-1 enables a negative feedback response when bound to its ligand, which is absent in NIB1412-activated CD4^+^ T cells and thus could lead to dysregulated functional outputs.

### PD-1 engagement reduces phospho PTEN levels in T_EMs_ stimulated by anti-CD3 but not CD28SA

The engagement of CD28 on T cells leads to the activation of the phosphatidylinositol 3-kinase (PI3K)/ protein kinase B (Akt) pathway which is important for cell survival and growth.^17^ Phosphatase and tensin homolog (PTEN), a lipid phosphatase, negatively regulates the PI3K/Akt pathway.^18^ A recent study has shown that the engagement of the PD-1 pathway suppresses casein kinase 2 (CK2) and results in the inhibition of the stabilizing-phosphorylation of the Ser380-Thr382-Thr383 residue cluster of PTEN. This increases PTEN phosphatase activity which subsequently inhibits the phosphatidylinositol 3-kinase (PI3K)/Akt pathway.^19^ Our results show that T_EMs_ stimulated with NIB1412 have significantly higher pPTEN levels compared with when stimulated with anti-CD3 mAb. T_EMs_ stimulated with anti-CD3 in the presence of rPD-L1 result in significantly diminished pPTEN compared with the anti-CD3 alone condition (**Fig. 6****A**). The rPD-L1 & NIB1412 condition showed similarly high pPTEN levels compared with the NIB1412 alone condition (**Fig. 6****A**). Consistent with the previous result, CK2 expression was found to be significantly higher in T_EMs_ activated with NIB1412 than when stimulated with anti-CD3 mAb (**Fig. 6****B**). The CK2 levels, although not significant, was reduced in T_EMs_ stimulated with anti-CD3 in the presence of rPD-L1, but no reduction was observed in the rPD-L1+NIB1412 condition. These results point to the lack of engagement of the PD-1 mediated regulation of the PI3K-PTEN-CK2 signaling axis in CD28SA-activated T cells.

## Discussion

TGN1412 is a CD28SA that caused an uncontrolled expansion of T cells with an accompanying cascade of proinflammatory cytokine release and infiltration of T cells into tissues, in all six healthy volunteers enrolled in a phase I clinical study.^3,4^ Despite initial recovery, their conditions deteriorated and this may be linked to a prolonged adverse response due to the dysregulation of the immune system by TGN1412.

We show that prolonged stimulatory signals, upregulation of the CCR5 receptor and the LFA-1 integrin on the cell surface, and lack of inhibitory receptors such as PD-1 in CD28SA-treated T cells are responsible for the hyperactive phenotype observed. We also showed the prolonged unrestricted proliferation in CD28SA-activated effector memory CD4^+^ T cells. These results indicate the unrestricted expansion of a CD28SA-induced immune response, while the anti-CD3-induced immune response contracts post-activation. Enhanced and prolonged secretion of IL-2 and autocrine triggering of IL-2 receptors (IL-2Rs) could contribute to sustained proliferation of CD28SA-stimulated T cells. Data from our lab indicate that NIB1412-simulated T cell proliferation requires IL-2-IL-2R interaction (unpublished results). The contraction phase of T cell activation is mainly brought about through apoptosis induced via the Fas-FasL system,^20^ and we have shown a diminished capacity of CD28SA stimulated T cells to upregulate Fas (CD95), which might explain the failure of these T cells to undergo normal contraction.

Effector memory cells respond to the inflammatory chemokines CCL3, CCL4 and CCL5, and upon re-stimulation upregulate CCR5 and become tissue invasive.^21^ TGN1412 can induce monocytes to secrete high levels of CCL5 within 2 h of stimulation.^22^ It is likely that CD28SA-induced upregulation of CCR5 was a contributing factor to the lymphocyte tissue invasion observed in the volunteers during the TGN1412 clinical trial. A prominent feature of the pathologic events in the trial patients was prolonged lymphocyte depletion from the circulation which was suggested to be related to unregulated T cell extravasation.^3,15^ LFA-1 is the predominant adhesion molecule on T cells^23^ and plays an important role in extravasation of lymphocytes into inflamed tissue. Since NIB1412-stimulated T_EMs_ expressed high levels of LFA-1, it is possible that these cells might be more likely to undergo extravasation. We show enhanced adhesion and transendothelial migration of NIB1412-stimulated T_EMs_ compared with anti-CD3-stimulated T_EMs_. A variety of negative regulators are known to play roles in the resolution of T cell response with PD-1 being a key inhibitory receptor.^6^ The PD-1 pathway has been shown to play crucial roles in the regulation of autoimmunity, transplantation immunity, infectious immunity, and tumor immunity.^7^ The PD-1-PD-L1 interaction plays an important role during the contraction phase of the immune response.^7^ PD-1 deficient experimental mouse models display a breakdown of tolerance and a higher susceptibility to proliferate with higher amounts of IFN-γ production,^24^ which leads to the development of spontaneous strain-dependent autoimmune diseases. The recognition that the PD-1 pathway provides strong negative signaling and is a dominant regulator of activated T cells has led to focus on development of therapeutic strategies aimed at manipulating this PD-1.^7^ Thus, PD-1 was chosen as a candidate co-inhibitory receptor to see its potential lack of contribution in CD28SA-activated T cells. In our study, we show that CD28SA-activated T cells fail to upregulate PD-1 and the negative feedback conferred by the PD-1-PD-L1 interaction was found to be absent in these T cells. Further investigations are required in order to gain an understanding of the mechanisms that regulate PD-1 expression and function in this context.

Although, PD-1 was absent on the surface of CD28SA-activated CD4^+^ T cells, it is present within the cytoplasm of these cells. PD-1 has been shown to be present in vesicles near the Golgi, and in the trans-Golgi network.^25^ Since PD-1 is known to be upregulated on the cell surface upon TCR triggering, it suggests that the fusion of PD-1-containing vesicles within the cytoplasm to the plasma membrane is dependent on signaling pathways that emanate from the TCR that are not engaged by CD28SA. The exact molecular signals that connect TCR to PD-1 upregulation require further definition.

We have shown that engagement of the PD-1 receptor by PD-L1 reduces PTEN phosphorylation in anti-CD3-stimulated T_EMs_ but not in CD28A-stimulated T_EMs_. The presence of phosphorylated PTEN in CD28SA-stimulated T_EMs_ suggests that there is an absence of PTEN lipid phosphatase activity to inhibit the PI3K/AKT signaling axis, leaving the T cells in a hyper-proliferative state. Since the PI3K pathway regulates diverse cellular activities such as growth, migration, survival, vesicular trafficking and glucose metabolism,^26^ it would be worth investigating key signaling proteins that branch from the PI3K nexus that may be dysregulated during CD28SA activation.

The lack of a reliable biomarker predictive of uncontrolled T cell stimulation by TGN1412 was a key factor in the failure of preclinical safety testing of this immunostimulatory mAb. Detailed understanding of the target biology followed by identification and screening of target specific biomarkers for risk assessment is essential to improve the safety profile of biologics.^27^ Our study demonstrates that CD28SA-stimulated T cells display a phenotype that is reflective of a predisposition to excessive activation and lack of regulation by PD-1 mediated inhibitory signals, which can potentially be used as biomarkers to predict the effects a T cell stimulatory biologic. However, the utility of testing for functionality of the PD-1 pathway as a biomarker of hazard needs further investigation and validation.

## Methods

### Reagents

All reagents were obtained from Sigma-Aldrich unless otherwise stated.

### T cell isolation

Ethical approval for the use of human peripheral blood mononuclear cells (PBMCs) from healthy donors was approved by the local ethical committee and all subjects provided informed consent. PBMCs were isolated from heparinised venous blood by density gradient separation (Lymphoprep, Axis-Shield, cat # О7811). The effector memory CD4^+^ T cell (T_EM_) isolation kit (Miltenyi Biotec, cat # 130–094–125) was used to purify T_EMs_ from PBMCs according to manufacturer's instructions.

### Endothelial cell culture

Human dermal microvascular endothelial cells (HDMEC) were purchased from Promocell and were cultured in endothelial cell basal media (EBM) MV2 growth media (C-22221; Promocell), supplemented with 5% (v/v) fetal calf serum (FCS) and EGF (5 ng/ml), VEGF (0.5 ng/ml), FGF-2 (10 ng/ml), long R3 insulin growth factor-1 (20 ng/ml), hydrocortisone (0.2 μg/ml) and ascorbic acid (1 μg/ml) (supplement pack C-39221; Promocell).

### Stimulating antibodies and recombinant proteins

Humanized superagonistic anti-CD28 antibody NIB1412, a human IgG4 sharing the H chain V region and L chain sequences of TGN1412,^28^ was generated at the National Institute for Biological Standards and Control (NIBSC). Murine anti-human CD3 (clone: UCHT1, cat # 16–0038–85) antibody was purchased from eBioscience. The cross-linking goat anti-human IgG (Fc) secondary antibody was purchased from AbD Serotec (cat # 5211–8004). Recombinant PD-L1 was purchased from R&D Systems (cat # 156-B7–100).

### Proliferation assays

96-well round-bottom non-tissue culture treated plates were coated with stimulating antibodies at 37°C for 2 h. Plates were washed twice to remove unbound antibody before addition of T cells. Where NIB1412 was used, the wells were pre-coated with cross-linking goat anti-human IgG (Fc) mAb anti-IgG mAb (20 μg/ml). T cells were cultured in complete media (RPMI 1640 supplemented with 15% fetal calf serum (Life Technologies), 2 mM L-glutamine, 50 U ml^−1^ penicillin and 0.05 mg ml^−1^ streptomycin) for 72 h (37 °C in 5% CO_2_ at 95% humidity), and 18 h before the end of the time point, the cells were pulsed with tritiated thymidine ([^3^H]-TdR, 0.5 μCi/well,). Incorporation of [^3^H]-TdR in T cells was determined using a β-scintillation counter (MicroBetaTrilux; PerkinElmer Life Sciences,). Data obtained are presented as mean counts per minute (cpm).

### Flow cytometric analysis

T cells were stained for 20 min at 4°C using the following fluorochrome conjugated antibodies (eBioscience) diluted to the optimal concentration: anti–CD4 PerCP-Cyanine5.5 (RPA-T4, cat # 45–0049–42), anti–PD-1-PE (J105, cat # 12–2799–42), anti–CD11a-FITC (HI111, cat # 11–0119–42), anti-CCR5-PE (NP-6G4, cat # 12–1956–42), anti–TCR-APC (IP26, cat # 17–9986–42) and anti–IFNγ-APC (4S.B3, cat # 17–7319–82). For intracellular staining, surface markers were stained first, followed by permeabilization using Cytofix/Cytoperm (BD PharMingen, cat # 555028) and stained with anti–IFNγ-APC or anti–PD-1-PE. Fluorescent signals from cells were acquired on BD FACS Canto II flow cytometer and data were analyzed using Cyflogic software v. 1.2.1.

### Cell cycle analysis

Cells stimulated and cultured as described above were collected at the end of each time point, fixed in ice-cold 70% ethanol and kept at 4°C until analysis. Fixed cells were washed and treated with RNase for 20 min. Subsequently, 5 μg/mL of propidium iodide and 0.1% Triton-X were added for 15 min and analyzed by flow cytometry.

### Measurement of IL-2

IL-2 levels in culture supernatants were determined by enzyme-linked immunosorbent assay (ELISA) using the Human Ready-SET-Go! ELISA kit from eBioscience (cat # 88–7025–22) following manufacturer's instructions.

### Re-stimulation assay using rPD-L1

Isolated T cells were stimulated and cultured as described above for 48 h and re-stimulated in a 96-well round-bottom non-tissue culture treated plate pre-coated with 1 μg/ml anti-CD3, 10 μg/ml rPD-L1 and the combination of 1 μg/ml anti-CD3 + 10 μg/ml rPD-L1. Cells were incubated at 37°C for one hour after which Brefeldin A (GolgiPlug; BD Biosciences, cat# 555029) was added and incubated for another 5 h. Intracellular IFNγ staining was then performed and cells were analyzed using flow cytometry as described above.

### Gel Electrophoresis and Western Immunoblotting

Cells were lysed with NP40 lysis buffer and 20 μg of protein lysate was resolved by 10–12% SDS-PAGE, transferred to PVDF membranes (Bio-Rad), blocked, and probed with the primary antibodies: anti-pPTEN (phospho S380+ T382+T383) antibody (Abcam, cat # ab131107) and anti-casein kinase IIα (1AD9) (Santa Cruz Biotechnology, cat # sc-12738) followed by appropriate horseradish peroxidase-conjugated secondary antibodies (Cell Signaling Technology) and visualized using the ECL system (PerkinElmer Life Sciences).

### Static adhesion and transendothelial migration of T_EMs_

Isolated T_EMs_ were stimulated with 5 μg/ml anti-CD3, 10μg/ml NIB1412 and the CD3+NIB1412 as described above and harvested after 48 h. Cells were incubated with 1 μM of CellTracker™ Green CMFDA (Molecular Probes) according to manufactures instructions. For the adhesion assay HDMECs were seeded on gelatin-coated 96-well tissue culture plates (Nunc) in endothelial cell media for 48 h, while for the transendothelial migration assay HDMECs were seeded on gelatin-coated Transwell inserts (6.5 mm diameter, 3.0 μm polyester membrane; Corning Costar). Media was then changed to 1% (v/v) FCS containing media for 24 h. For the adhesion assay the CMFDA-labeled T_EMs_ (0.2 mL, 5 × 10^5^ cells/mL) were added to the confluent HDMECs and incubated for 30 min. After washing three times with PBS (0.2 mL), cells were fixed and stained with Alexa 568-conjugated phalloidin (Invitrogen®, cat # A-12380) and Hoechst 33342 (Invitrogen®, cat # H-21492) before imaging. Adherent cells in three randomly selected optical fields per well were visualized, counted and images were acquired using a Axio Observer Zeiss microscope with objective LD “Plan-Neofluar” 20x/0.4 Corr Ph2 M27, and analyzed with ZEN Pro 2012 software. For the transendothelial cell migration assay, CMFDA-labeled T_EMs_ (1 × 10^5^ cells/insert) were added to the confluent HDMECs and incubated for 12 h. Cells in the lower compartment were collected and fluorescence was quantified using a Varioskan™ Flash Multimode Plate Reader (Thermo Scientific).

### Statistical analysis

Unpaired *t* test was used to analyze proliferation, adhesion, transendothelial migration, PTEN and CK2 densitometric data and results presented as mean ± SD/SEM. Two-way ANOVA followed by Bonferroni test was used to analyze IL-2 ELISA data using GraphPad Prism 5 (GraphPad Software Inc.). *P* values of <0.05 were considered to be statistically significant.
